# Supplementary material for: Immune profiling of mpox survivors reveals divergent durability of antibody and T cell responses
Source: Nat Commun. 2025 Dec 4;17:570. doi: 10.1038/s41467-025-67266-7 (PMC12808142; doi:10.1038/s41467-025-67266-7)
Supplement: Supplementary file 1 — Supplementary Information [file 41467_2025_67266_MOESM1_ESM.pdf]

Supplementary information

Immune profiling of mpox survivors reveals divergent durability of antibody and T cell responses

Supplementary Fig 1-14
Supplementary Table 1

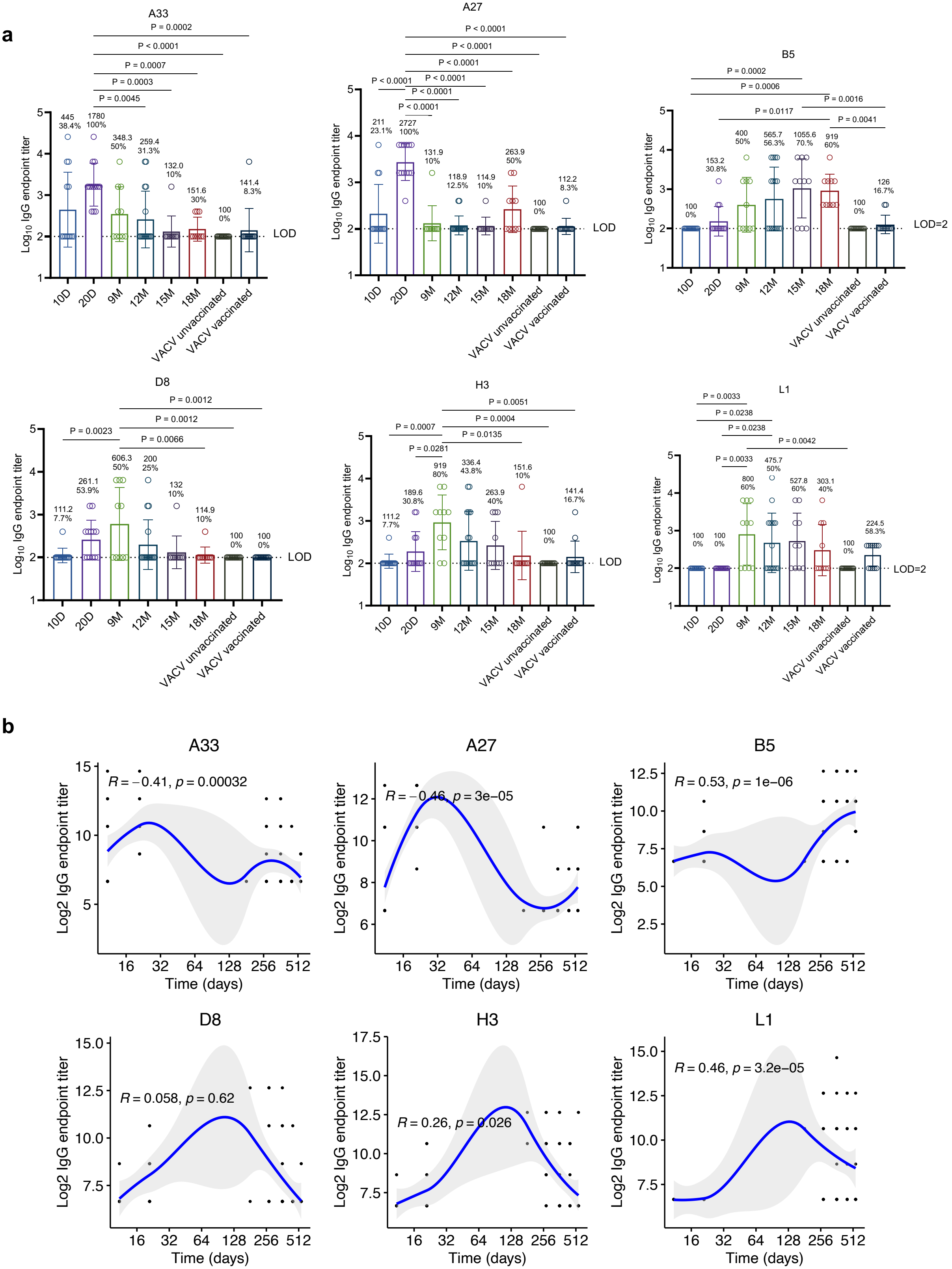

a
A33
P = 0.0002
P < 0.0001
P = 0.0007
P = 0.0003
P = 0.0045
445 38.4%
1780 100%
348.3 50%
259.4 31.3%
132.0 10%
151.6 30%
100 0%
141.4 8.3%
LOD
Log10 IgG endpoint titer
A27
P < 0.0001
P < 0.0001
P < 0.0001
P < 0.0001
P < 0.0001
P < 0.0001
P < 0.0001
211 23.1%
2727 100%
131.9 10%
118.9 12.5%
114.9 10%
263.9 50%
100 0%
112.2 8.3%
LOD
B5
P = 0.0002
P = 0.0006
P = 0.0117
P = 0.0016
P = 0.0041
100 0%
153.2 30.8%
400 50%
565.7 56.3%
1055.6 70.%
919 60%
100 0%
126 16.7%
LOD=2
10D
20D
9M
12M
15M
18M
VACV unvaccinated
VACV vaccinated
D8
P = 0.0012
P = 0.0012
P = 0.0023
P = 0.0066
111.2 7.7%
261.1 53.9%
606.3 50%
200 25%
132 10%
114.9 10%
100 0%
100 0%
LOD
H3
P = 0.0051
P = 0.0004
P = 0.0007
P = 0.0135
P = 0.0281
111.2 7.7%
189.6 30.8%
919 80%
336.4 43.8%
263.9 40%
151.6 10%
100 0%
141.4 16.7%
LOD
L1
P = 0.0033
P = 0.0238
P = 0.0238
P = 0.0033
P = 0.0042
100 0%
100 0%
800 60%
475.7 50%
527.8 60%
303.1 40%
100 0%
224.5 58.3%
LOD=2
b
A33
R = −0.41, p = 0.00032
A27
R = −0.46, p = 3e−05
B5
R = 0.53, p = 1e−06
D8
R = 0.058, p = 0.62
H3
R = 0.26, p = 0.026
L1
R = 0.46, p = 3.2e−05
Log2 IgG endpoint titer
Time (days)
16
32
64
128
256
512

Supplementary Fig 1. Kinetics of plasma IgG responses to VACV antigens up to 18 months post-recovery. (a) Plasma IgG responses against major VACV membrane proteins were measured by ELISA in all enrolled participants. Mean endpoint titers and seropositivity rates are indicated in each panel. Four participant groups were included in this study: (1) acute-phase mpox patients, from whom blood samples were collected at 10 days (n=13) and 20 days (n=13) after symptom onset; (2) mpox convalescents who were recruited and followed for up to 18 months post-onset (9M, n=10; 12M, n=16; 15M, n=10; 18M, n=10); (3) a VACV-unvaccinated cohort (n=30); and (4) a VACV-vaccinated cohort (n=30). Mean endpoint titers and seropositivity rates are indicated above each column. All assays were conducted with 3 technical replicates per sample. Statistical significance was evaluated using one-way ANOVA followed by Tukey's multiple comparisons test. All analyses were conducted as two-sided. Error bars represent mean $\pm$ SD. Statistical significance is denoted as: ns ($p > 0.05$), *$p < 0.05$, **$p < 0.01$, ***$p < 0.001$, ****$p < 0.0001$. Only comparisons with p values ≤ 0.05 are shown. (b) The kinetics of VACV-specific IgG endpoint titers over an 18-month period post-recovery were visualized using scatter plots with locally estimated scatterplot smoothing (LOESS). Blue lines represent the fitted curve obtained using the LOESS curve fitting polynomial regression, and the gray band areas represent 95% confidence intervals. Pearson correlation coefficients and two-sided corresponding p-values were computed and displayed on the plots.

a

MPXV protein	Ratio (10D)	Ratio (20D)	Ratio (6M)	Ratio (9M)	Ratio (12M)	Ratio (15M)	Ratio (18M)
A29	46.15%	100.00%	0.00%	20.00%	6.25%	10.00%	20.00%
A35	38.46%	100.00%	100.00%	30.00%	50.00%	50.00%	70.00%
M1	7.69%	30.77%	50.00%	30.00%	18.75%	20.00%	30.00%
B6	61.54%	100.00%	0.00%	50.00%	56.25%	70.00%	60.00%
E8	84.62%	100.00%	50.00%	70.00%	62.50%	90.00%	60.00%
H3	30.77%	53.85%	100.00%	40.00%	62.50%	80.00%	40.00%

b

VACV protein	Ratio (10D)	Ratio (20D)	Ratio (6M)	Ratio (9M)	Ratio (12M)	Ratio (15M)	Ratio (18M)
A27	23.08%	100.00%	0.00%	10.00%	12.50%	10.00%	50.00%
A33	38.46%	100.00%	0.00%	50.00%	31.25%	10.00%	30.00%
L1	0.00%	0.00%	100.00%	60.00%	50.00%	60.00%	40.00%
B5	0.00%	30.77%	0.00%	50.00%	56.25%	70.00%	60.00%
D8	7.69%	53.85%	50.00%	50.00%	25.00%	10.00%	10.00%
H3	7.69%	30.77%	100.00%	80.00%	43.75%	40.00%	10.00%

Supplementary Fig 2. Seropositivity rates of IgG antibodies against major MPXV and VACV antigens. (a) Seropositivity rates of IgG antibodies targeting MPXV antigens. (b) Seropositivity rates of IgG antibodies targeting VACV antigens.

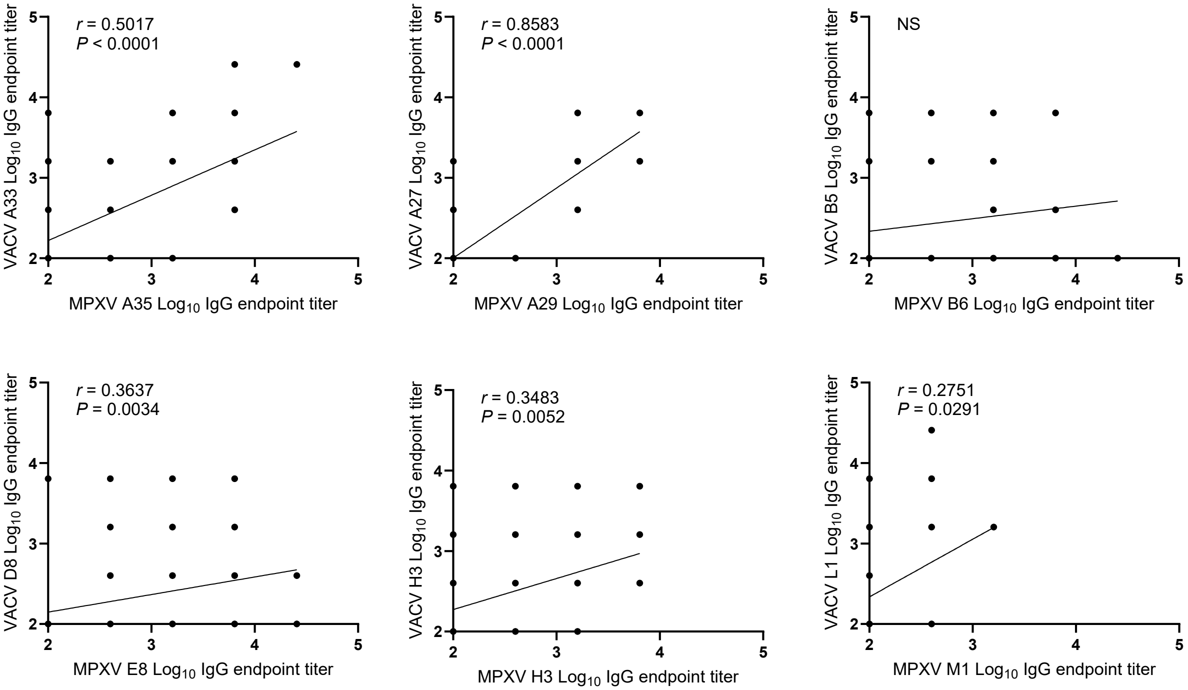

Supplementary Fig 3. Correlation analysis of IgG binding responses to MPXV (A29, A35, M1, B6, E8, H3) and VACV (A33, A27, L1, B5, D8, H3) antigens in MPXV convalescent individuals. Correlation analysis of IgG binding responses to MPXV (A29, A35, M1, B6, E8, H3) and VACV (A33, A27, L1, B5, D8, H3) antigens in MPXV convalescent individuals was performed using two-sided Pearson's correlation tests. Correlation coefficients (r) and corresponding p values are shown, and the color scale indicates the strength and direction of the correlation between antigen-specific IgG responses.

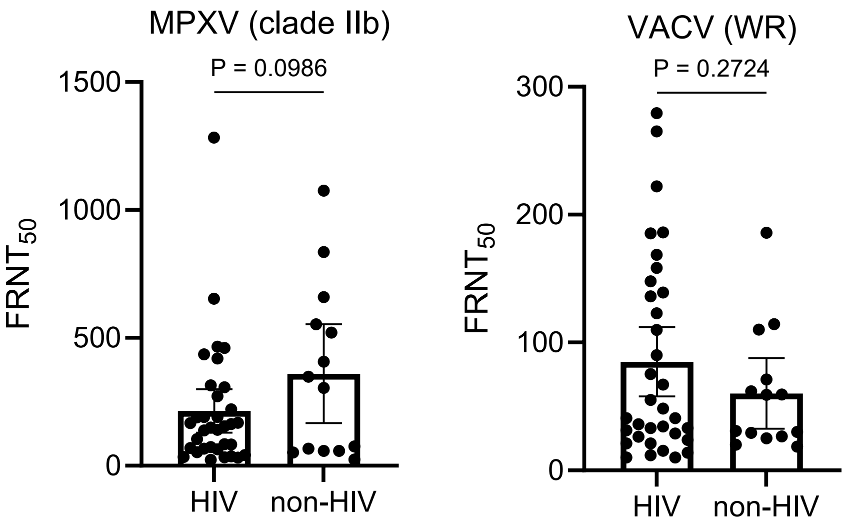

Supplementary Fig 4. Impact of HIV infection on neutralizing antibody responses in MPXV convalescents. (a) Neutralizing antibody titers ($FRNT_{50}$) against MPXV clade IIb in convalescent individuals, stratified by HIV infection status. (b) Neutralizing antibody titers against VACV (WR strain) in the same cohorts. All assays were conducted with 3 technical replicates per sample. No significant differences were observed between HIV-positive and HIV-negative participants. Horizontal bars indicate median values; statistical comparisons were performed using the Mann–Whitney U test. Error bars represent mean $\pm$ SD. Statistical significance is denoted as: ns ($P > 0.05$), *$P < 0.05$, **$P < 0.01$, ***$P < 0.001$, ****$P < 0.0001$.

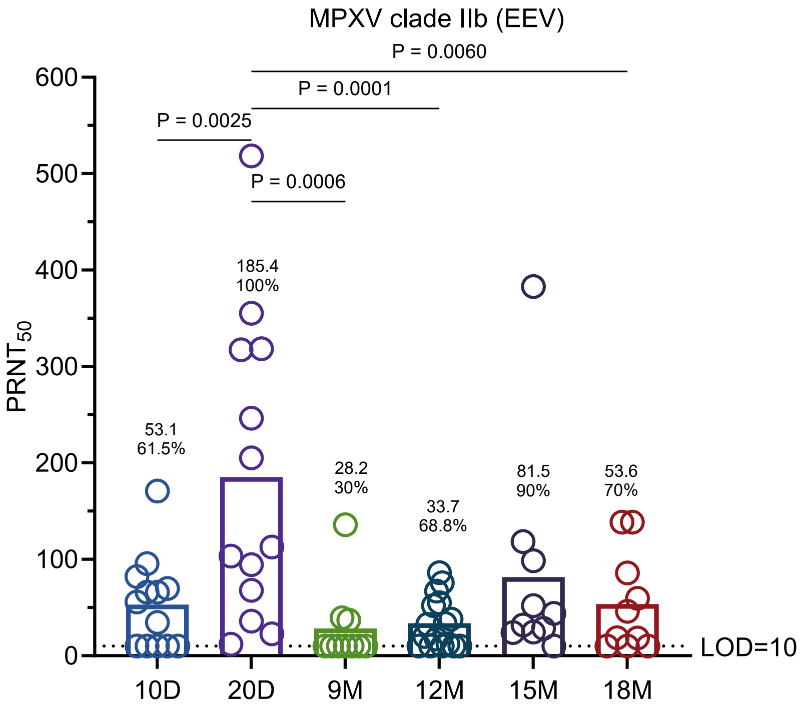

Supplementary Fig 5. Neutralization titers against MPXV clade IIb EEV form were determined by PRNT. Each column presents the mean endpoint titers, and rates of seropositivity. All assays were conducted with 3 technical replicates per sample. Statistical significance was evaluated using one-way ANOVA followed by Tukey's multiple comparisons test. All analyses were conducted as two-sided. Error bars represent mean ± SD. Statistical significance is denoted as: ns ($P > 0.05$), *$P < 0.05$, **$P < 0.01$, ***$P < 0.001$, ****$P < 0.0001$. Only comparisons with P values ≤ 0.05 are shown.

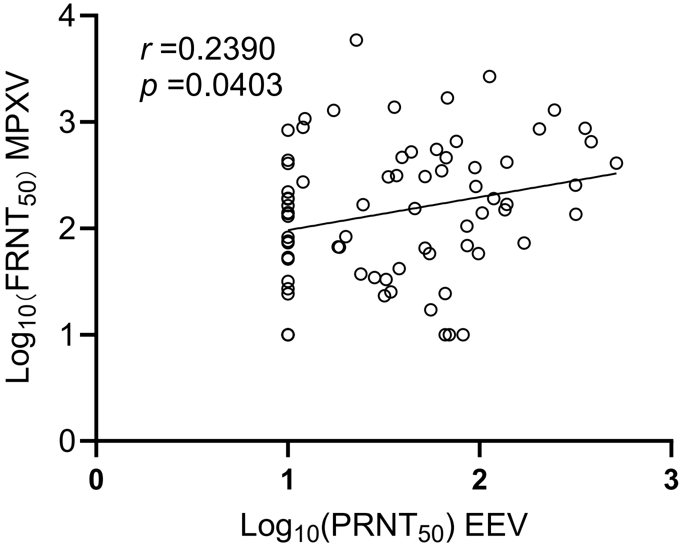

Supplementary Fig 6. Correlation between neutralizing titers against the MPXV mixture and the EEV form was assessed using two-sided Pearson's correlation tests. Correlation coefficients (r) and corresponding p values are shown, and the color scale indicates the strength and direction of the correlation.

MPXV clade IIb (NCBI No. NC_003310.1)	MPXV clade Ib (NCBI No. NC_063383.1)	VACV WR (NCBI No. NC_006998.1)	AA homology between clade IIb and clade Ib	AA homology between clade IIb and WR	AA homology Between clade Ib and WR
A29	A29	A27	98.2%	95.5%	95.5%
A35	A35	A33	98.9%	94.6%	94.6%
M1	M1	L1	100%	100%	100%
B6	B6	B5	100%	98.7%	98.7%
E8	E8	D8	99.7%	97.0%	96.7%
H3	H3	H3	99.4%	96.6%	96.6%
A21	A21	A21	99.1%	98.3%	97.4%
A17	A17	A16	99.2%	98.1%	97.3%
A30	A30	A28	100%	97.9%	97.9%
A28	A28	A26	97.3%	94.9%	93.5%
H2	H2	H2	100%	99.5%	99.5%
I2	I2	I2	100%	98.6%	98.6%
G2	G2	G2	100%	98.2%	98.2%
F7	F7	E8	99.6%	98.9%	98.5%

Supplementary Fig 7. Comparative sequence homology analysis of 14 major membrane-associated antigens across Monkeypox virus (MPXV) clade IIb, MPXV clade Ib, and Vaccinia virus (VACV) WR strain. The analysis includes key serological targets and structural components of the mature virion (MV) or enveloped virion (EV), comprising A29, A35, M1, B6, E8, H3, A21, A17, A30, A28, H2, I2, G2, and F7. Panels present pairwise amino-acid (AA) identity (%) across the three orthopoxvirus lineages. Full-length open reading frames from representative genomes of MPXV clade IIb, MPXV clade Ib, and VACV WR were aligned using MEGA, and sequence identity was calculated based on global amino-acid alignment across each complete coding region.

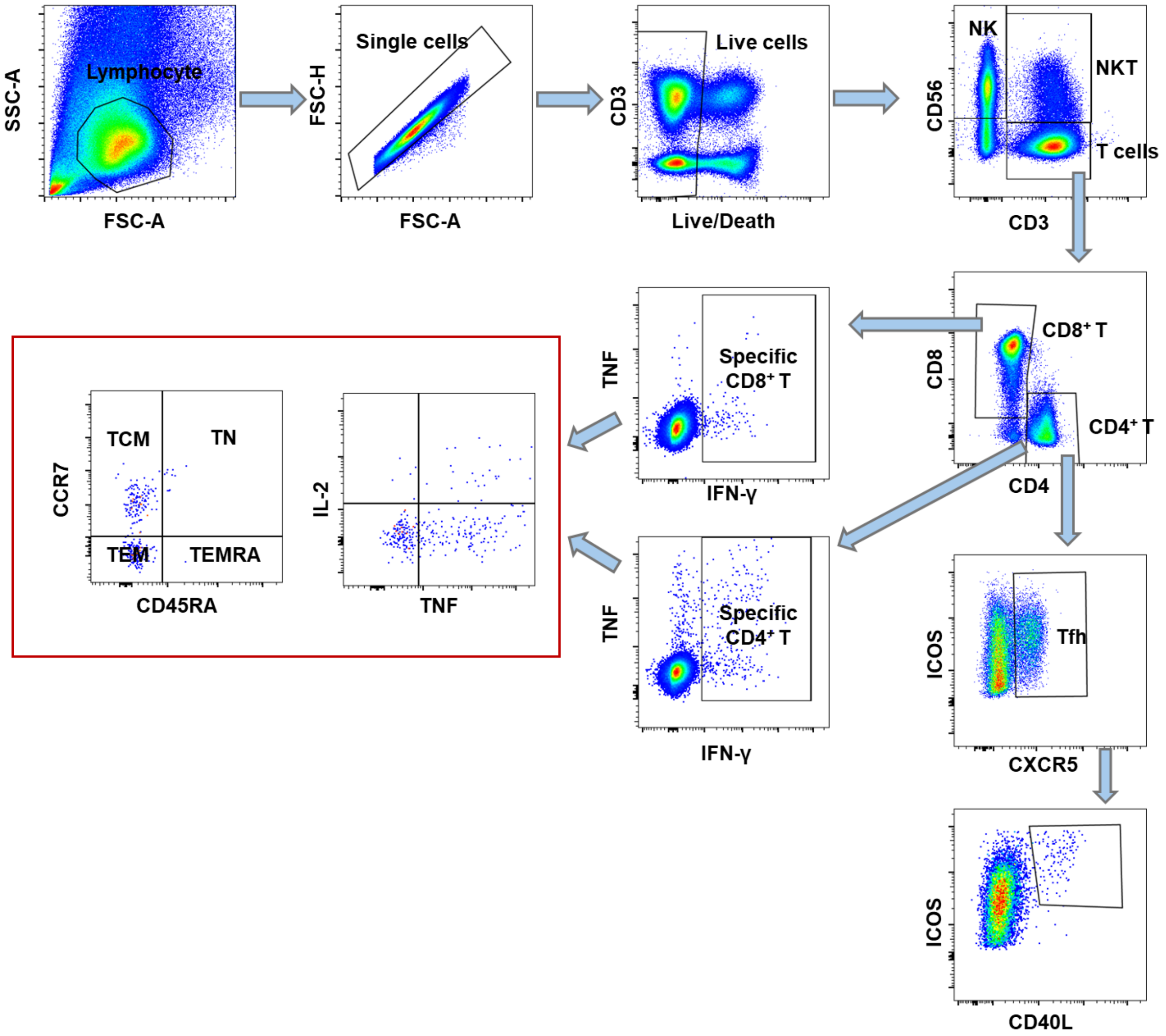

Supplementary Fig 8. Gating strategy for identifying CD4$^+$, CD8$^+$, and circulating T follicular helper (cTfh) T-cell subsets in MPXV convalescent individuals. Dead cells were excluded using a fixable viability dye, followed by surface staining with antibodies to CD3, CD4, CD8, CD56, CCR7, CXCR5, ICOS, and CD45RA for 30 min at room temperature. Cells were then fixed and permeabilized, and intracellular cytokine staining for CD40L, IFN-γ, TNF-α, and IL-2 was performed to assess MPXV-specific responses. Data were acquired on a BD FACSymphony™ S6 and analyzed using FlowJo v10.6.2. Antigen-specific responses were quantified within CD4$^+$, CD8$^+$, and cTfh (CD4$^+$CXCR5$^+$ICOS$^+$) subsets. A positive response was defined as an IFN-γ$^+$ T-cell frequency above background (stimulated minus unstimulated > 0).

a

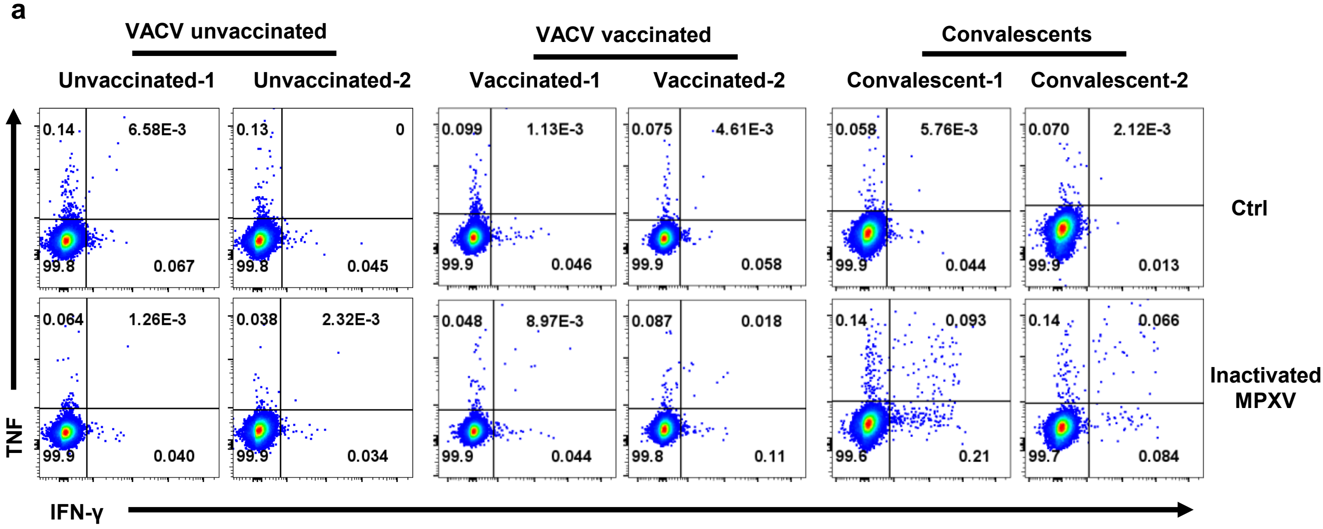

b

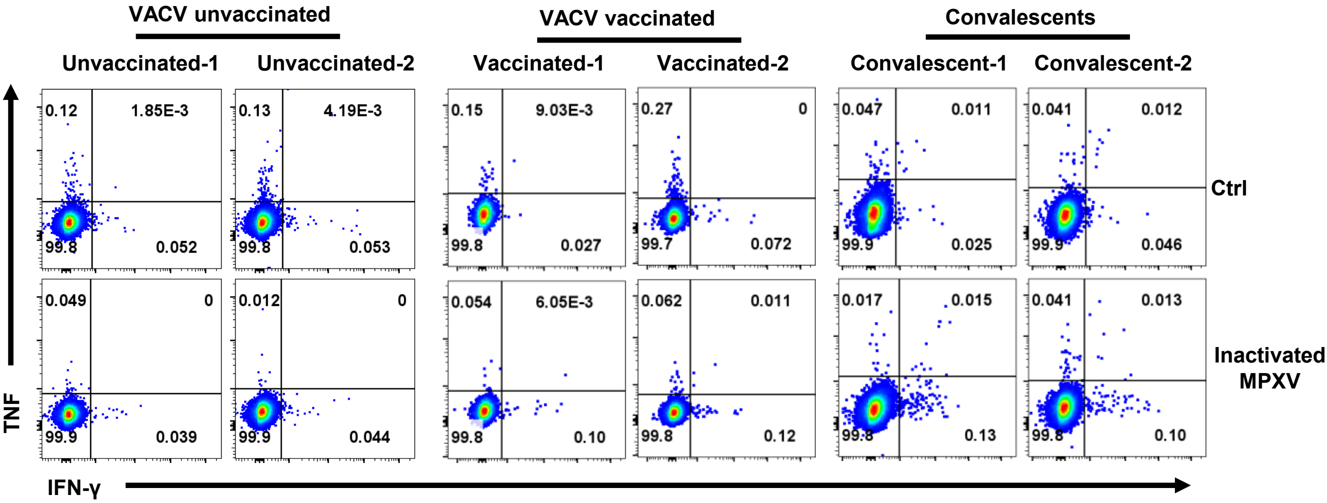

Supplementary Fig 9. Evaluation of MPXV-specific T-cell responses during the convalescent phase. Representative flow cytometry plots of antigen-specific CD4$^+$ (a) and CD8$^+$ (b) T cells are shown for the three cohorts: VACV-unvaccinated individuals, VACV-vaccinated individuals, and MPXV convalescents. Corresponding plots from mock-stimulated negative controls are also included.

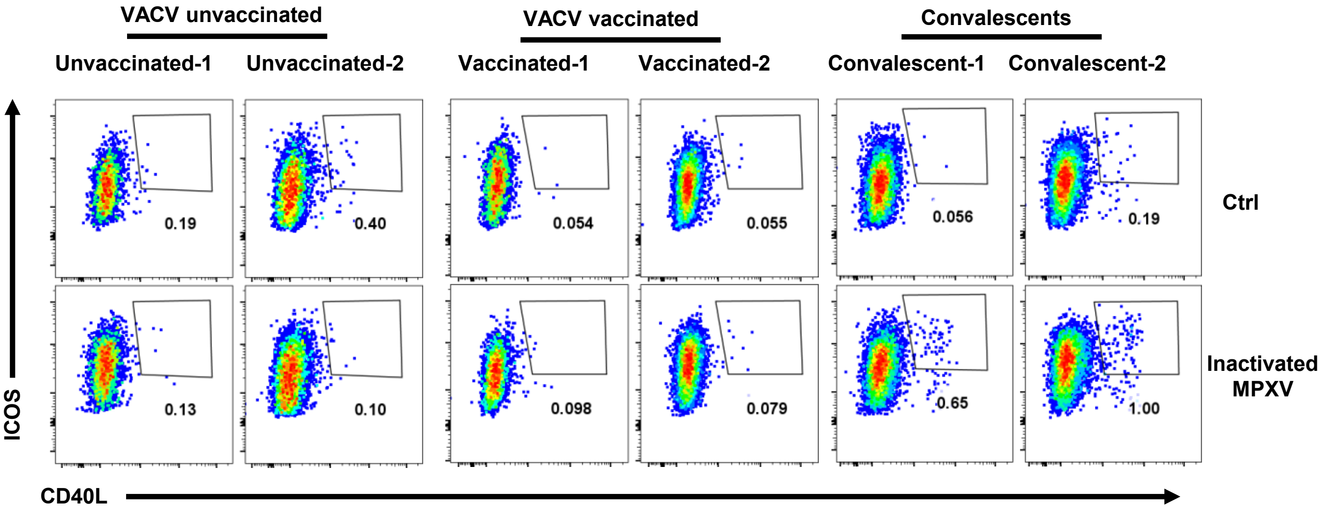

Supplementary Fig 10. Evaluation of MPXV-specific cTfh-cell responses during the convalescent phase. Representative flow plots are presented. Corresponding plots from mock-stimulated negative controls are also included.

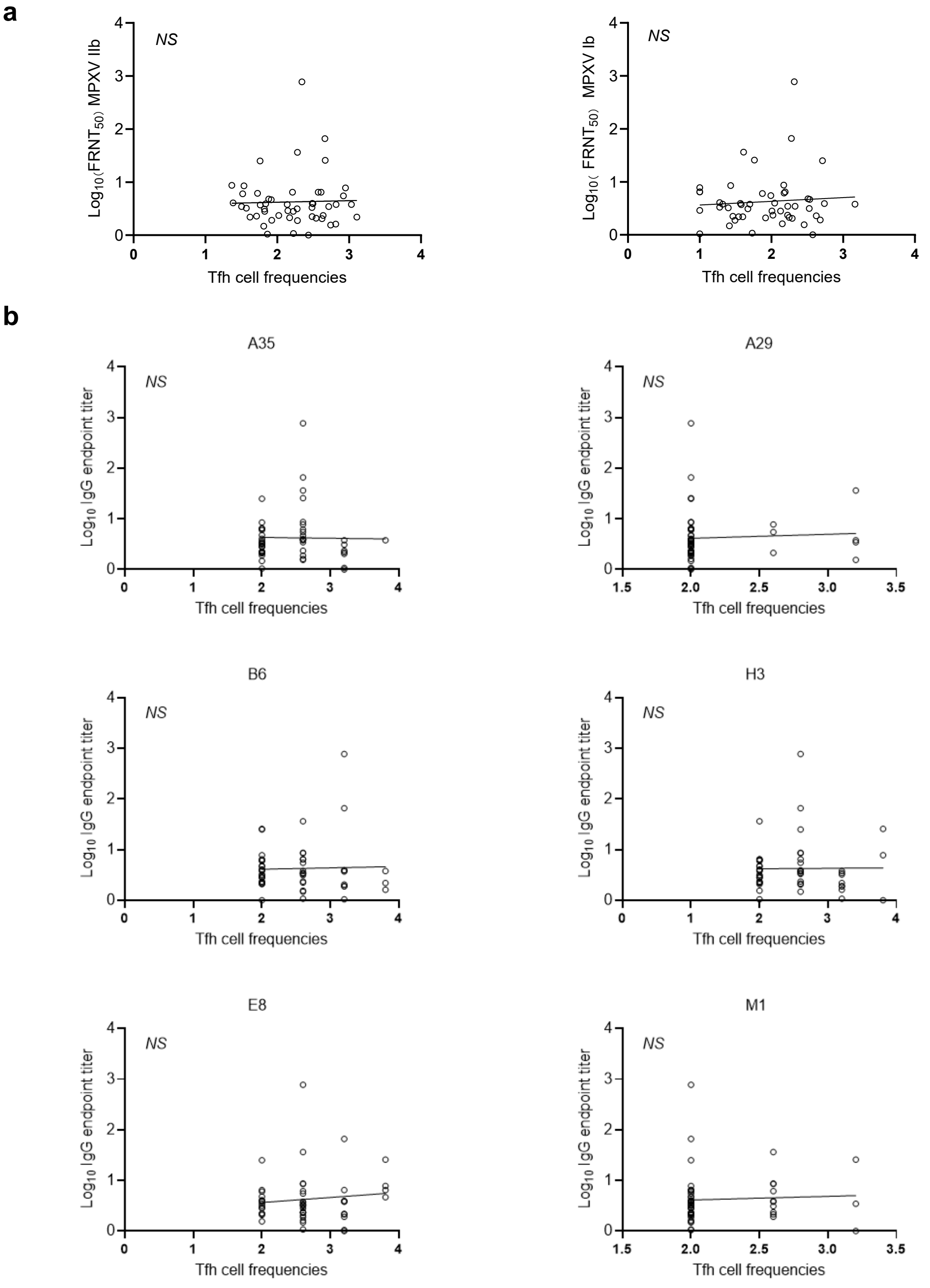

Supplementary Fig 11. Correlation of circulating Tfh cell frequencies with MPXV-specific antibody responses. Circulating Tfh cell frequencies were compared with MPXV-specific neutralizing antibody titers ($FRNT_{50}$) (a) and binding IgG titers (b) against MPXV surface antigens in convalescent individuals. No significant correlations were observed in either case. Each dot represents an individual participant. Correlations were assessed using two-sided Spearman's rank tests.

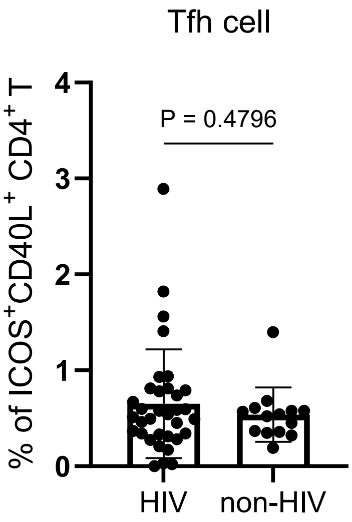

Supplementary Fig 12. Comparison of circulating Tfh cell frequencies between HIV-positive and HIV-negative MPXV convalescents. Circulating Tfh cell frequencies were quantified in PBMCs collected from convalescent individuals stratified by HIV status. All assays were conducted with 3 technical replicates per sample. No significant differences were observed between groups. Data are shown as individual values with median bars; statistical significance was assessed using the Mann–Whitney U test.

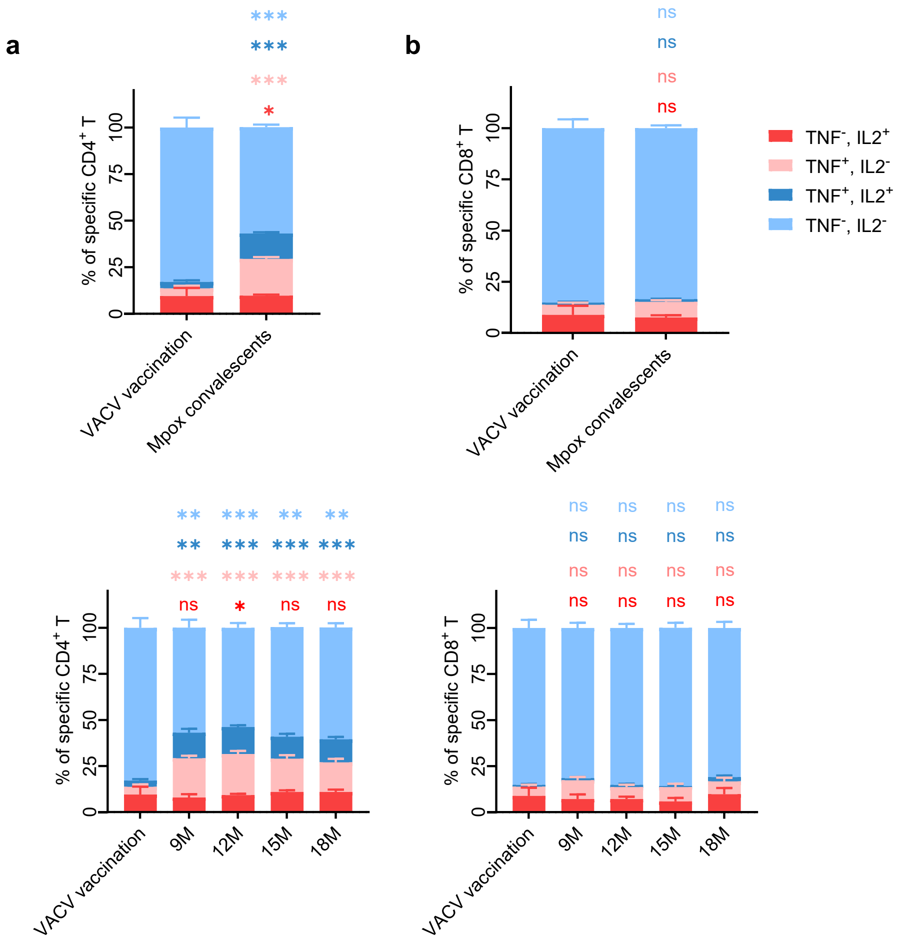

Supplementary Fig 13. Assessment of functional capacity of MPXV-specific T cells, cytokine production of TNF and IL-2 was evaluated. TNF-α and IL-2 production in antigen-stimulated $CD4^+$ (a) and $CD8^+$ T (b) cells, quantified after background subtraction (stimulated minus unstimulated), along with the corresponding temporal dynamics following MPXV infection recovery. The statistical symbols above the bar chart represent the results of statistical analyses comparing the populations corresponding to the colors of each group with those of the VACV vaccination group. All assays were conducted with 3 technical replicates per sample. Comparisons were performed using the Mann-Whitney test. P values are indicated as ns ($P > 0.05$), *$P < 0.05$, **$P < 0.01$, ***$P < 0.001$.

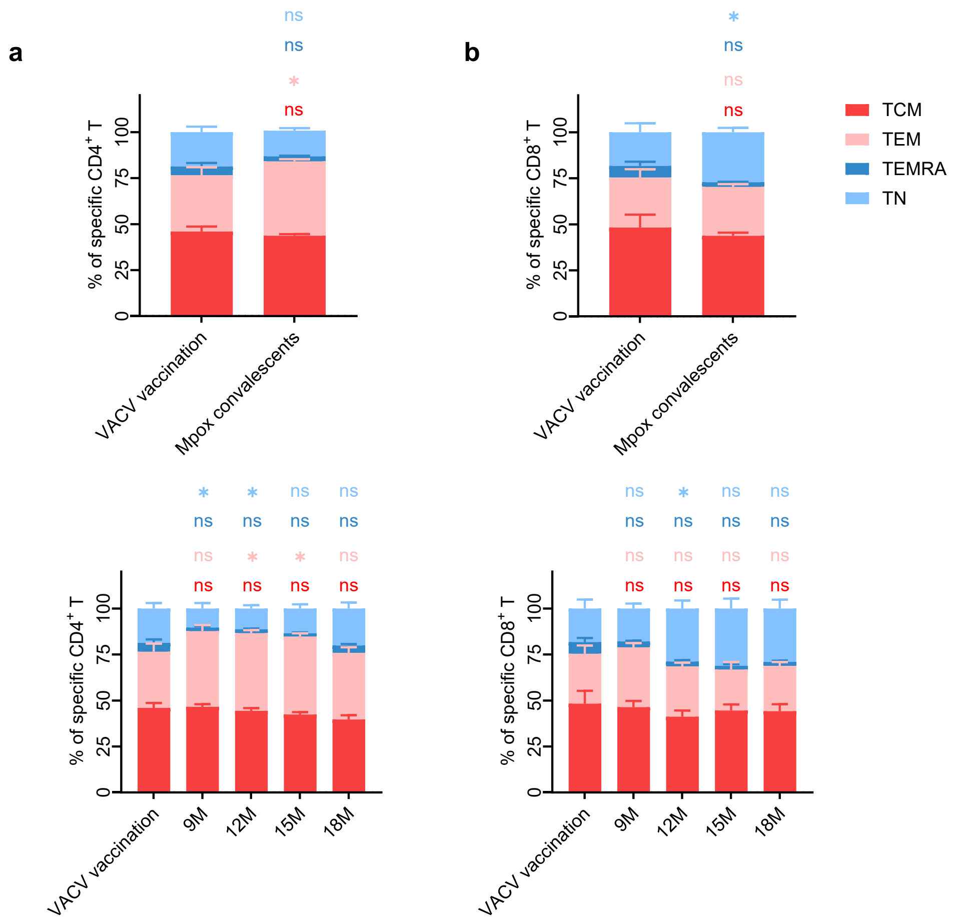

Supplementary Fig 14. Comparative profiling of CD4⁺ and CD8⁺ T cells subsets in MPXV convalescents revealed distinct distributions across naive T cells (TN), central memory T cells (TCM), effector memory T cells (TEM) and CD45RA⁺ effector memory T cells (TEMRA). (a) $CD4^+$ T-cell subset distribution, including naïve T cells (TN, $CCR7^+CD45RA^+$), central memory T cells (TCM, $CCR7^+CD45RA^-$), effector memory T cells (TEM, $CCR7^-CD45RA^-$), and $CD45RA^+$ effector memory T cells (TEMRA, $CCR7^-CD45RA^+$). The panel also shows temporal changes in $CD4^+$ subset composition across different convalescence time intervals. (b) $CD8^+$ T-cell subset distribution, similarly grouped into TN, TCM, TEM, and TEMRA subsets, along with the corresponding temporal dynamics following MPXV infection recovery. All assays were conducted with 3 technical replicates per sample. Comparisons were performed using the Mann-Whitney test. P values are indicated as ns ($P > 0.05$), *P < 0.05, **P < 0.01, ***P < 0.001.

Supplemental Table 1. Detailed information regarding participants enrolled in this study

Sample	Gender	Age	HIV infection	Genotypes of MPXV isolates	Sampling time
MPXV convalescent-1	Male	20-29	Negative	Clade IIb	12M
MPXV convalescent-2	Male	30-39	Positive	Clade IIb	12M, 15M, 18M
MPXV convalescent-3	Male	20-29	Positive	Clade IIb	12M, 15M, 18M
MPXV convalescent-4	Male	30-39	Positive	Clade IIb	15M, 18M
MPXV convalescent-5	Male	20-29	Negative	Clade IIb	12M, 15M, 18M
MPXV convalescent-6	Male	20-29	Positive	Clade IIb	12M, 15M, 18M
MPXV convalescent-7	Male	30-39	Positive	Clade IIb	12M, 15M, 18M
MPXV convalescent-8	Male	20-29	Positive	Clade IIb	12M, 15M, 18M
MPXV convalescent-9	Male	30-39	Negative	Clade IIb	12M
MPXV convalescent-10	Male	20-29	Positive	Clade IIb	12M
MPXV convalescent-11	Male	40-49	Positive	Clade IIb	12M, 15M, 18M
MPXV convalescent-12	Male	20-29	Negative	Clade IIb	12M, 15M, 18M
MPXV convalescent-13	Male	30-39	Positive	Clade IIb	9M, 12M
MPXV convalescent-14	Male	30-39	Negative	Clade IIb	9M, 12M
MPXV convalescent-15	Male	20-29	Positive	Clade IIb	12M
MPXV convalescent-16	Male	20-29	Positive	Clade IIb	9M, 12M
MPXV convalescent-17	Male	30-39	Negative	Clade IIb	9M, 12M
MPXV convalescent-18	Male	30-39	Positive	Clade IIb	9M, 15M, 18M
MPXV convalescent-19	Male	20-29	Positive	Clade IIb	9M
MPXV convalescent-20	Male	20-29	Negative	Clade IIb	9M
MPXV convalescent-21	Male	30-39	Negative	Clade IIb	6M, 9M
MPXV convalescent-22	Male	10-19	Positive	Clade IIb	6M, 9M
MPXV convalescent-23	Male	30-39	Positive	Clade IIb	9M
MPXV patient-1	Male	20-29	Negative	Clade IIb	10D, 20D
MPXV patient-2	Male	30-39	Negative	Clade IIb	10D, 20D
MPXV patient-3	Male	40-49	Negative	Clade IIb	10D, 20D
MPXV patient-4	Male	50-59	Negative	Clade IIb	10D, 20D
MPXV patient-5	Male	20-29	Negative	Clade IIb	10D, 20D
MPXV patient-6	Male	30-39	Negative	Clade IIb	10D, 20D
MPXV patient-7	Male	20-29	Negative	Clade IIb	10D, 20D
MPXV patient-8	Male	30-39	Negative	Clade IIb	10D, 20D
MPXV patient-9	Male	30-39	Negative	Clade IIb	10D, 20D
MPXV patient-10	Male	20-29	Negative	Clade IIb	10D
MPXV patient-11	Male	30-39	Negative	Clade IIb	10D
MPXV patient-12	Male	30-39	Negative	Clade IIb	10D
MPXV patient-13	Male	20-29	Negative	Clade IIb	10D
MPXV patient-14	Male	20-29	Negative	Clade IIb	20D
MPXV patient-15	Male	30-39	Negative	Clade IIb	20D
MPXV patient-16	Male	20-29	Negative	Clade IIb	20D
MPXV patient-17	Male	30-39	Negative	Clade IIb	20D
VACV vaccinated-1	Male	60-69	Negative	NA	NA
VACV vaccinated-2	Male	60-69	Negative	NA	NA
VACV vaccinated-3	Male	80-89	Negative	NA	NA
VACV vaccinated-4	Male	70-79	Negative	NA	NA
VACV vaccinated-5	Male	70-79	Negative	NA	NA
VACV vaccinated-6	Female	80-89	Negative	NA	NA
VACV vaccinated-7	Female	50-59	Negative	NA	NA
VACV vaccinated-8	Male	70-79	Negative	NA	NA
VACV vaccinated-9	Female	50-59	Negative	NA	NA
VACV vaccinated-10	Male	70-79	Negative	NA	NA
VACV vaccinated-11	Male	60-69	Negative	NA	NA
VACV vaccinated-12	Male	60-69	Negative	NA	NA
VACV vaccinated-13	Female	80-89	Negative	NA	NA
VACV vaccinated-14	Female	80-89	Negative	NA	NA
VACV vaccinated-15	Male	70-79	Negative	NA	NA
VACV vaccinated-16	Male	60-69	Negative	NA	NA
VACV vaccinated-17	Male	60-69	Negative	NA	NA
VACV vaccinated-18	Male	60-69	Negative	NA	NA
VACV vaccinated-19	Female	60-69	Negative	NA	NA
VACV vaccinated-20	Female	60-69	Negative	NA	NA

VACV vaccinated-21	Male	60-69	Negative	NA	NA
VACV vaccinated-22	Male	60-69	Negative	NA	NA
VACV vaccinated-23	Male	60-69	Negative	NA	NA
VACV vaccinated-24	Male	60-69	Negative	NA	NA
VACV vaccinated-25	Male	60-69	Negative	NA	NA
VACV vaccinated-26	Male	60-69	Negative	NA	NA
VACV vaccinated-27	Male	70-79	Negative	NA	NA
VACV vaccinated-28	Female	60-69	Negative	NA	NA
VACV vaccinated-29	Male	70-79	Negative	NA	NA
VACV vaccinated-30	Male	70-79	Negative	NA	NA
VACV unvaccinated-1	Male	30-34	Negative	NA	NA
VACV unvaccinated-2	Female	30-39	Negative	NA	NA
VACV unvaccinated-3	Male	20-29	Negative	NA	NA
VACV unvaccinated-4	Female	30-39	Negative	NA	NA
VACV unvaccinated-5	Female	30-39	Negative	NA	NA
VACV unvaccinated-6	Female	30-39	Negative	NA	NA
VACV unvaccinated-7	Female	30-39	Negative	NA	NA
VACV unvaccinated-8	Female	20-29	Negative	NA	NA
VACV unvaccinated-9	Male	30-39	Negative	NA	NA
VACV unvaccinated-10	Female	30-39	Negative	NA	NA
VACV unvaccinated-11	Female	30-39	Negative	NA	NA
VACV unvaccinated-12	Female	30-39	Negative	NA	NA
VACV unvaccinated-13	Male	40-49	Negative	NA	NA
VACV unvaccinated-14	Male	20-29	Negative	NA	NA
VACV unvaccinated-15	Female	30-39	Negative	NA	NA
VACV unvaccinated-16	Female	30-39	Negative	NA	NA
VACV unvaccinated-17	Female	30-39	Negative	NA	NA
VACV unvaccinated-18	Female	20-29	Negative	NA	NA
VACV unvaccinated-19	Female	20-29	Negative	NA	NA
VACV unvaccinated-20	Female	30-39	Negative	NA	NA
VACV unvaccinated-21	Female	30-39	Negative	NA	NA
VACV unvaccinated-22	Female	30-39	Negative	NA	NA
VACV unvaccinated-23	Female	30-39	Negative	NA	NA
VACV unvaccinated-24	Female	30-39	Negative	NA	NA
VACV unvaccinated-25	Female	30-39	Negative	NA	NA
VACV unvaccinated-26	Female	30-39	Negative	NA	NA
VACV unvaccinated-27	Female	30-39	Negative	NA	NA
VACV unvaccinated-28	Female	30-39	Negative	NA	NA
VACV unvaccinated-29	Male	20-29	Negative	NA	NA
VACV unvaccinated-30	Male	20-29	Negative	NA	NA

NA not available, M month, D day
