# Supplementary material for: Immune profiling of mpox survivors reveals divergent durability of antibody and T cell responses
Source: Nat Commun. 2025 Dec 4;17:570. doi: 10.1038/s41467-025-67266-7 (PMC12808142; doi:10.1038/s41467-025-67266-7)
Supplement: Supplementary file 3 — Reporting Summary [file 41467_2025_67266_MOESM3_ESM.pdf]

nature portfolio

Corresponding author(s): Jincun Zhao

Last updated by author(s): Sep 12, 2025

Reporting Summary

Nature Portfolio wishes to improve the reproducibility of the work that we publish. This form provides structure for consistency and transparency in reporting. For further information on Nature Portfolio policies, see our Editorial Policies and the Editorial Policy Checklist.

Statistics

For all statistical analyses, confirm that the following items are present in the figure legend, table legend, main text, or Methods section.

n/a	Confirmed	
☐	☒	The exact sample size (*n*) for each experimental group/condition, given as a discrete number and unit of measurement
☐	☒	A statement on whether measurements were taken from distinct samples or whether the same sample was measured repeatedly
☐	☒	The statistical test(s) used AND whether they are one- or two-sided<br>*Only common tests should be described solely by name; describe more complex techniques in the Methods section.*
☒	☐	A description of all covariates tested
☒	☐	A description of any assumptions or corrections, such as tests of normality and adjustment for multiple comparisons
☐	☒	A full description of the statistical parameters including central tendency (e.g. means) or other basic estimates (e.g. regression coefficient) AND variation (e.g. standard deviation) or associated estimates of uncertainty (e.g. confidence intervals)
☐	☒	For null hypothesis testing, the test statistic (e.g. *F*, *t*, *r*) with confidence intervals, effect sizes, degrees of freedom and *P* value noted<br>*Give P values as exact values whenever suitable.*
☒	☐	For Bayesian analysis, information on the choice of priors and Markov chain Monte Carlo settings
☒	☐	For hierarchical and complex designs, identification of the appropriate level for tests and full reporting of outcomes
☐	☒	Estimates of effect sizes (e.g. Cohen's *d*, Pearson's *r*), indicating how they were calculated

Our web collection on statistics for biologists contains articles on many of the points above.

Software and code

Policy information about availability of computer code

Data collection	No software was used for data collection.
Data analysis	We used GraphPad Prism 8.0 for the analysis of the data. FlowJo v10.6.2 was used to analyze Flow cytometry data.

For manuscripts utilizing custom algorithms or software that are central to the research but not yet described in published literature, software must be made available to editors and reviewers. We strongly encourage code deposition in a community repository (e.g. GitHub). See the Nature Portfolio guidelines for submitting code & software for further information.

Data

Policy information about availability of data

All manuscripts must include a data availability statement. This statement should provide the following information, where applicable:

- Accession codes, unique identifiers, or web links for publicly available datasets
- A description of any restrictions on data availability
- For clinical datasets or third party data, please ensure that the statement adheres to our policy

All numeral data produced in this study are included as source data. Any other data that support the findings of this study are available from the corresponding author upon request.

Research involving human participants, their data, or biological material

Policy information about studies with human participants or human data. See also policy information about sex, gender (identity/presentation), and sexual orientation and race, ethnicity and racism.

Reporting on sex and gender	Information on participants' sex and gender was collected and provided in the supplementary materials; however, no gender-specific analyses were conducted.
Reporting on race, ethnicity, or other socially relevant groupings	All participants included in this study were residents of Guangzhou, China. Information on race or ethnicity was not collected.
Population characteristics	A total of 40 individuals with PCR-confirmed monkeypox virus (MPXV) infection were enrolled between August 2022 and January 2023 to investigate antibody responses. All infections were attributed to MPXV clade IIb, with 37.5% (15/40) of participants living with HIV. The median age was 30 years, and all participants self-identified as men who have sex with men (MSM). None had received smallpox vaccination within the past decade . All individuals developed symptomatic disease, characterized by fever and rash, with no severe cases or fatalities reported. Participants were followed longitudinally for up to 18 months post-onset of symptoms (POS), with samples collected at approximately 10 and 20 days, and at 6, 9, 12, 15, and 18 months, depending on availability. For comparative analysis, we also included 30 age-matched healthy controls and 30 individuals with historical smallpox vaccination (>30 years prior) .
Recruitment	We conducted a prospective, longitudinal cohort study to characterize immune responses in individuals recovering from MPXV infection. The study was initiated at the Guangzhou Center for Disease Control and Prevention (Guangzhou, China), with participant enrolment beginning in 2023.
Ethics oversight	The study protocol was approved by the institutional ethics committees of Guangzhou CDC (GZCDC-ECHR-2023P0059) and Shenzhen Third People's Hospital (2021-030). Written informed consent was obtained from all participants prior to enrolment.

Note that full information on the approval of the study protocol must also be provided in the manuscript.

Field-specific reporting

Please select the one below that is the best fit for your research. If you are not sure, read the appropriate sections before making your selection.

☒ Life sciences ☐ Behavioural & social sciences ☐ Ecological, evolutionary & environmental sciences

For a reference copy of the document with all sections, see nature.com/documents/nr-reporting-summary-flat.pdf

Life sciences study design

All studies must disclose on these points even when the disclosure is negative.

Sample size	A total of 40 individuals with PCR-confirmed monkeypox virus (MPXV) infection were enrolled between August 2022 and October 2024 to investigate antibody responses.Participants were followed longitudinally for up to 18 months post-onset of symptoms (POS), with samples collected at approximately 10 and 20 days, and at 6, 9, 12, 15, and 18 months, depending on availability. For comparative analysis, we also included 30 age-matched healthy controls and 30 individuals with historical smallpox vaccination (>30 years prior).
Data exclusions	No data was excluded from analysis.
Replication	Experiments had at least three, independent, biological replicates and each biological replicate had three technical replicates. All attempts at replication were successful.
Randomization	No specific steps were taken to randomize experimental groups. This is because the classification of patients is clear before recruitment.
Blinding	Blinding was not relevant to our study because the infectious status of the MPXV-infected patients has been verified.

Reporting for specific materials, systems and methods

We require information from authors about some types of materials, experimental systems and methods used in many studies. Here, indicate whether each material, system or method listed is relevant to your study. If you are not sure if a list item applies to your research, read the appropriate section before selecting a response.

Materials & experimental systems

n/a	Involved in the study
☐	☒ Antibodies
☐	☒ Eukaryotic cell lines
☒	☐ Palaeontology and archaeology
☒	☐ Animals and other organisms
☒	☐ Clinical data
☒	☐ Dual use research of concern
☒	☐ Plants

Methods

n/a	Involved in the study
☒	☐ ChIP-seq
☐	☒ Flow cytometry
☒	☐ MRI-based neuroimaging

Antibodies

Antibodies used: True-Stain Monocyte Blocker™ (Biolegend, 426103), Human TruStain FcX™ (Biolegend, 422302), CD3-BUV395 (BD, 563546), CD4-BV510 (BD, 562970), CD8-AF647 (Biolegend, 344726), CD56-BV650 (BD, 564057), CXCR5-PerCP-Cy5.5(BD, 562781), CD45RA-BUV737 (BD, 612846), CCR7-APC-Fire750 (Biolegend, 353246), ICOS-BB515 (BD, 564549), CD40L-PE (Biolegend, 310806), IFN-γ-BV421 (Biolegend, 506538), TNF-α-PE-Cy7 (Biolegend, 502930), IL-2-BV605 (BD, 564165) and Fixable Viability Stain 440UV (BD, 566332).

Validation: All antibodies were commercially available. Validation statements and citations, available on the manufacturer's websites

Eukaryotic cell lines

Policy information about cell lines and Sex and Gender in Research

Cell line source(s): Vero 81 (CCL81), Vero E6 (CRL-1586) and RK-13 (CCL-37) cells were obtained from the American Type Culture Collection (ATCC). All cell lines were grown in DMEM (Gibco) supplemented with 10% fetal bovine serum (FBS, Gibco), and tested to be mycoplasma-free.

Authentication: None of the cell lines used were authenticated.

Mycoplasma contamination: All cell lines used were tested (by PCR) and were mycoplasma free.

Commonly misidentified lines (See ICLAC register): No commonly misidentified lines were used in this study.

Plants

Seed stocks: na

Novel plant genotypes: na

Authentication: na

Flow Cytometry

Plots

Confirm that:

☒ The axis labels state the marker and fluorochrome used (e.g. CD4-FITC).

☒ The axis scales are clearly visible. Include numbers along axes only for bottom left plot of group (a 'group' is an analysis of identical markers).

☒ All plots are contour plots with outliers or pseudocolor plots.

☒ A numerical value for number of cells or percentage (with statistics) is provided.

Methodology

Sample preparation: Peripheral blood mononuclear cells (PBMCs) were isolated from MPXV convalescent individuals and seeded at 5×10^5 to 1×10^6 cells per well in 96-well round-bottom plates. To evaluate MPXV-specific T-cell immunity, PBMCs were stimulated with heat-inactivated MPXV (56°C for 30 minutes) at 37°C, 16–18 hours later, brefeldin A (BioLegend) were supplemented to inhibit cytokine secretion with another 6 hours incubation. After stimulation, cells were washed and incubated at room

temperature with True-Stain Monocyte Blocker™ and Human TruStain FcX™ for 10 minutes to reduce nonspecific antibody binding. Dead cells were excluded using a viability dye, followed by surface staining with antibodies targeting CD3, CD4, CD8, CD56, CCR7, CXCR5, ICOS and CD45RA at room temperature for 30 minutes. Cells were then fixed and permeabilized using reagents from BD Biosciences, followed by intracellular cytokine staining for CD40L, IFN-γ, TNF, and IL-2 to profile MPXV-specific T-cell responses.

Instrument	Data acquisition was performed on a BD FACSymphony™ S6.
Software	Analyses were conducted using FlowJo software (v10.6.2; BD Biosciences).
Cell population abundance	Ex vivo analysis, no sorted cells were implicated in this study.
Gating strategy	Dead cells were excluded using a viability dye, followed by surface staining with antibodies targeting CD3, CD4, CD8, CD56, CCR7, CXCR5, ICOS and CD45RA at room temperature for 30 minutes. Cells were then fixed and permeabilized using reagents from BD Biosciences, followed by intracellular cytokine staining for CD40L, IFN-γ, TNF, and IL-2 to profile MPXV-specific T-cell responses.

☒ Tick this box to confirm that a figure exemplifying the gating strategy is provided in the Supplementary Information.
